# Chest high-resolution computed tomography can make higher accurate stages for thoracic sarcoidosis than X-ray

**DOI:** 10.1186/s12890-022-01942-y

**Published:** 2022-04-16

**Authors:** Yuan Zhang, Shan-shan Du, Meng-meng Zhao, Qiu-hong Li, Ying Zhou, Jia-cui Song, Tao Chen, Jing-yun Shi, Bing Jie, Wei Li, Li Shen, Fen Zhang, Yi-liang Su, Yang Hu, Elyse E. Lower, Robert P. Baughman, Huiping Li

**Affiliations:** 1 Department of Respiratory Medicine, School of Medicine, Shanghai Pulmonary Hospital, Tongji University, 507 Zheng Min Road, Shanghai, 200433 China; 2 Department of Radiology, School of Medicine, Shanghai Pulmonary Hospital, Tongji University, Shanghai, China; 3 Department of Internal Medicine, University of Cincinnati Medical Center, 1001 Holmes, 200 Albert Sabin Way, Cincinnati, OH 45267 USA

**Keywords:** Sarcoidosis, Pulmonary, Chest x-ray, High-resolution computed tomography, Pleural involvement

## Abstract

**Background:**

To explore if chest high-resolution computed tomography (HRCT) can make higher accurate stages for thoracic sarcoidosis stage than X-ray (CRX) only.

**Methods:**

Clinical data from medical records of consecutive patients with a confirmed diagnosis of pulmonary sarcoidosis at Shanghai Pulmonary Hospital from January 1 2012 to December 31 2016 and consecutive patients treated at the Sarcoidosis Center of University of Cincinnati Medical Center, Ohio, USA from January 1 2010 to December 31 2015 were reviewed. The clinical records of 227 patients diagnosed with sarcoidosis (140 Chinese and 87 American) were reviewed. Their sarcoidosis stage was determined by three thoracic radiologists based on CXR and HRCT presentations, respectively. The stage determined from CXR was compared with that determined from HRCT.

**Results:**

Overall, 50.2% patients showed discordant sarcoidosis stage between CXR and HRCT (52.9% in Chinese and 44.8% in American, respectively). The primary reason for inconsistent stage between CXR and HRCT was failure to detect mediastinal lymph node enlargement in the shadow of the heart in CXR (22.1%) and small nodules because of the limited resolution of CXR (56.6%). Stage determined from HRCT negatively correlated with carbon monoxide diffusing capacity (DLCO) significantly (*P* < .01) but stage determined from CXR did not. Pleural involvement was detected by HRCT in 58 (25.6%) patients but only in 17 patients (7.5%) by CXR. Patients with pleural involvement had significantly lower forced vital capacity and DLCO than patients without it (both *P* < .05).

**Conclusion:**

Revised staging criteria based on HRCT presentations included 5 stages with subtypes in the presence of pleural involvement were proposed. Thoracic sarcoidosis can be staged more accurately based on chest HRCT presentations than based on CXR presentations. Pleural involvement can be detected more accurately by HRCT.

**Supplementary Information:**

The online version contains supplementary material available at 10.1186/s12890-022-01942-y.

## Background

Abnormal chest imaging presentations are often the primary clinical characteristics of thoracic sarcoidosis. Currently, thoracic sarcoidosis is staged based on chest radiography (Chest X-ray, CXR) presentations following the 1999 American Thoracic Society/European Respiratory Society/World Association of Sarcoidosis and other Granulomatous Disorders guidelines for sarcoidosis, which describe five stages of CXR presentations [1]. However, the diagnostic accuracy based on CXR presentations is only 50% [1–3].

The resolution of CXR is often too low to accurately detect small lung nodules, thin patchy lesions, and pleural involvement [4, 5]. In addition, CXR may fail to reveal mediastinal lymph node enlargement and pleural involvement that are in the shadow areas of the heart and diaphragm. Chest high-resolution computed tomography (HRCT) has a higher resolution and provides clear views of the pulmonary hilum, mediastinal lymph node, lung, and pleura, and thus has been successfully used to examine interstitial lung diseases (ILD) and small lung lesions [4–6]. Therefore, HRCT overcomes the limitations of CXR and can more accurately stage thoracic sarcoidosis than CXR. Furthermore, sarcoidosis CXR presentations have been found to be inconsistent with clinical stage [7], whereas sarcoidosis lesion characteristics presented on HRCT are consistent with pulmonary functional changes [8]. Despite the apparent benefits of HRCT over CXR, large scale multicenter studies to compare sarcoidosis stage determined based on CXR presentations versus based on HRCT presentations are still lacking. The current study aimed to fill this knowledge gap. Here, we compared sarcoidosis stage determined based on the presentations of the two examinations in 227 patients (140 Chinese and 87 American). Our results suggest that HRCT can more accurately stage thoracic sarcoidosis than CXR.

## Methods

### Study design

This was a retrospective observational study. The study protocols were approved by the Institutional Review Boards of Shanghai Pulmonary Hospital (Approval No. k15-189) and University of Cincinnati Medical Center (Approval No. 2013-3320). Clinical data from medical records of consecutive patients with a confirmed diagnosis of pulmonary sarcoidosis at Shanghai Pulmonary Hospital from January 1 2012 to December 31 2016 and consecutive patients treated at the Sarcoidosis Center of University of Cincinnati Medical Center, Ohio, USA from January 1 2010 to December 31 2015 were reviewed. Written consent to participate was obtained from all of the patients. The authors report no conflicts of interest.

### Patient inclusion and exclusion criteria

The inclusion criteria were: (1) histopathological analysis confirmed that the lesion specimens showed noncaseating epithelioid granuloma; (2) the diagnosis of sarcoidosis was confirmed. The histopathology of lymph node or subcutaneous nodules biopsy of the patients with sarcoidosis was characterized by non-caseous, non-necrotizing granulomas and showed negative TB, fungus and/or parasitic infection, tumor, and vasculitis. Additionally, to further confirm sarcoidosis, smear-negative TB was excluded based on the methods we had established [9, 10]; (3) patients had chest imaging data for at least one HRCT and one CXR, and the time between HRCT and CXR was less than two weeks. Patients with tuberculous, fungal and/or parasitic infections, and/or malignancy were excluded.

### CXR and HRCT evaluation

Three thoracic radiologists and two pulmonologists independently reviewed patients’ imaging data and staged sarcoidosis based on CXR and HRCT characteristics, respectively. Inconsistent staging results among the five specialists were discussed until a consensus was achieved. Sarcoidosis stage determined from CXR presentations was compared with that determined from HRCT presentations.

### Statistical analysis

The statistical analysis software SPSS 16.0 was used. Measurement variables are presented as mean (standard deviation, SD). Chi-square test was used to compare the differences in sarcoidosis stage of Chinese patients versus American patients. The association between sarcoidosis stage and lung functional parameters were analyzed by bivariate spearman correlation analysis. The difference in lung functional parameters of two patient groups was analyzed by independent *t*-test. *P* < 0.05 was considered statistically significant.

## Results

### Comparison of general clinical data of Chinese versus American patients

**Fig. 1:**
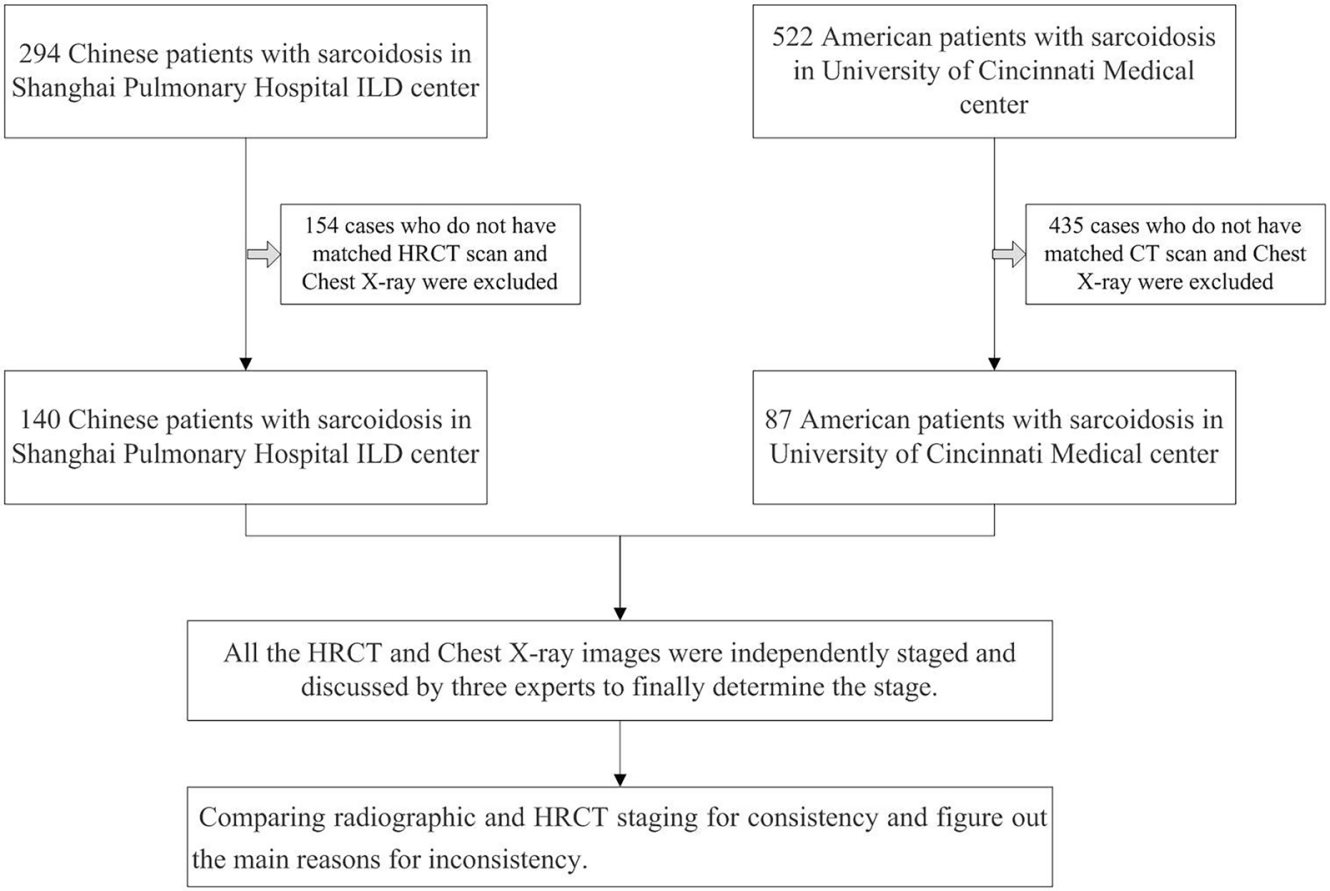
Patient flow chart

Patient selection flow chart is displayed in Fig. 1. A total of 227 subjects including 140 Chinese Han and 87 American patients (W/B, 31/56) with sarcoidosis were included in the analysis. Comparison of the general clinical data revealed significant differences in sarcoidosis stage in the two patient groups (*P* < 0.01, Table 2). Compared with the American patients, Chinese patients had substantially higher proportions of stage I (38.6% vs. 18.4%) and II (40% vs.12.6%) but lower proportion of stage IV (1.4% vs. 41.4%, Table 1). In addition, the Chinese patients showed significantly higher FVC, FVC%, FEV1, FEV1/FVC, DLCO, and DLCO% at the diagnosis (All *P* < 0.01, Table 1) than the American patients, suggesting that the Chinese patients might have better lung function at the diagnosis.

**Table 1:** Clinical characteristic of 227 patients at the diagnosis

Characteristics	Chinese (n = 140)	American (n = 87)
Mean age ± SD (years)	48.8 ± 8.1	43.6 + 11.7
Men/women	43/97 (0.44:1)	31/56 (0.55:1)
Race	Han (140)	Black/White (56/31)
**Sarcoidosis stage according to chest X-ray presentations**^**$**^**, n (%)**		
0	9 (6.4)	7 (8)
I	54 (38.6)	16 (18.4)
II	56 (40)	11 (12.6)
III	19 (13.6)	17 (19.5)
IV	2 (1.4)	36 (41.4)
**Sarcoidosis diagnosis by different approach (n)**		
EOB	48	41
Surgery	83	18
Extra lung biopsy	9	28
**Pulmonary function tests**		
FVC^$^, mean ± SD (L)	3.0 ± 0.7	2.6 ± 0.6
FVC^$^, predicted, mean ± SD (%)	84.7 ± 10.3	76.0 ± 16.4
FEV1^$^, mean ± SD (L)	2.4 ± 0.6	2.0 ± 0.6
FEV1/FVC^$^, mean ± SD	79.9 ± 5.9	71.8 ± 12.7
DLCO^$^, mean ± SD (L)	20.3 ± 4.6	15.2 ± 3.8
DLCO^$^, mean ± SD (%)	92.4 ± 12.2	63.9 ± 19.1

*SD* standard deviation, *EOB* electronic bronchoscope, *FVC* forced vital capacity, *FEV1* forced expiratory volume in one second, *DLCO* carbon monoxide diffusing capacity. Chi-square test was used for the comparison

^$^*p* < 0.01

### Comparison of sarcoidosis stage determined from CXR presentations versus from HRCT presentations

Sarcoidosis stage determined from CXR and HRCT presentations were not consistent (Table 3). Of the 227 patients, although the 67 patients at stage II and 38 at stage IV showed consistent sarcoidosis stage from the presentations of the two examinations, the consistence rates for the patients at stage 0, I and III were only 0%, 11.4% and 16.7%, respectively. The overall consistent rate in the227 patients was 50.2% (47.1% in Chinese and 55.2 in American) (Table 2).

**Table 2:** Comparison of sarcoidosis stage determined according to chest X-ray (CXR) versus chest HRCT presentations

Stage	Stage according to CXR (n)	Stage according to HRCT (n)				Consistent cases (n)	Consistency rate (%)
		I	II	III	IV		
**Total, n = 227**							
0	16	2	11	3	0	0	0.0
I	70	8	62	0	0	8	11.4
II	67	0	62	0	5	62	92.5
III	36	0	23	6	7	6	16.7
IV	38	0	0	0	38	38	100.0
Total	227	10	158	9	52	114	50.2
**Chinese patients, n = 140**							
0	9	2	7	0	0	0	0.0
I	54	6	48	0	0	6	11.1
II	56	0	56	0	0	56	100.0
III	19	0	15	2	2	2	10.5
IV	2	0	0	0	2	2	100.0
Total	140	8	126	2	4	66	47.1
**American patients, n = 87**							
0	7	0	4	3	0	0	0.0
I	16	2	14	0	0	2	12.5
II	11	0	6	0	5	6	54.5
III	17	0	8	4	5	4	23.5
IV	36	0	0	0	36	36	100.0
Total	87	2	32	7	46	48	55.2

### Reasons for the inconsistent sarcoidosis stage

We identified four reasons to explain the stage inconsistency from the two examinations (Additional file 2: Table S1). The most common reason in both patient groups was B1, which describes missed lesions by CXR because of the low resolution of CXR (Additional file 2: Table S1). The other reasons included missed lesions by CXR because the lesion imaging signals were masked by the heart and other intrathoracic structure in CXR (Additional file 2: Table S1). Lung fibrosis was not detected by CXR in patients at stage IV.

### Patients at stage 0 determined from CXR presentations

Sixteen patients presented respiratory symptoms but showed normal CXR, and thus were diagnosed as stage 0. Two of them presented mediastinal lymph node enlargement on the followed HRCT and were then moved to stage I (Additional file 1: Figure S1A and B), and the other 11 presented mediastinal lymph node enlargement combined with small lung nodules on HRCT and thus were moved to stage II (Additional file 1: Figure S1C, D, and E). Three of them were then moved to stage III based on their HRCT presentations (Additional file 1: Figure S1F and G). The reasons for missing the lesions were the low resolution of CXR and the locations of the lesions in the shadow areas of the heart or other thoracic structures (Additional file 2: Table S1).

### Patients at stage I determined from CXR presentations

Only 8 of the 70 patients (11.4%) at stage I determined from their CXR presentations remained at stage I based on their HRCT presentations. The other 62 patients (88.6%) only presented hilar enlargement on CXR, whereas showed additional intrathoracic lymph node enlargement and lung infiltrating opacities on their HRCT. Thus, the 62 patients were moved to stage II based on their HRCT presentations. (Additional file 1: Figure S2). These findings indicate that CXR may fail to reveal mild infiltrating lesions in the lung because of its low resolution.

### Patients at stage II determined from CXR presentations

All the 67 patients at stage II based on CXR remained at stage II when their HRCT presentations were used to determine the stage, suggesting that HRCT could detect all the lesions that can be found by CXR. Notably, the number of patients with stage II determined from HRCT presentations (158 cases) was substantially higher than that based on CXR presentations (67 cases). HRCT may identify lesions that could be missed by CXR and thus patients diagnosed as stage I based on CXR may be moved to stage II or higher when their HRCT presentations were used for sarcoidosis staging. In contrast to the Chinese patients, only 6 (54.5%) of the 11 American patients originally at stage II based on CXR remained at stage II based on HRCT, and the other 5 (45.5%) were moved to stage IV. These results suggest that CXR may not show lung fibrosis as accurately as HRCT.

### Patients at stage III determined from CXR presentations

Of the 36 patients at stage III based on CXR, 63.9% (23/36) were down staged to sarcoidosis stage II based on HRCT (Table 2) because hilar or mediastinal lymph node enlargement were missed from CXR (Additional file 1: Figure S3). Seven became stage IV based on HRCT because CXR failed to show lung fibrotic lesions.

### Correlation between pulmonary function parameters and sarcoidosis stage

Sarcoidosis stage determined from HRCT presentations significantly correlated with, FEV1/FVC, carbon monoxide diffusing capacity (DLCO), and DLCO% negatively (All *P* < 0.001, Table 3), suggesting that patient pulmonary function may become worse as sarcoidosis progress to higher stage. In contrast to the stage from HRCT presentations, the stage from CXR presentations showed no significant correlation with DLCO and DLCO%.

**Table 3:** Correlation between sarcoidosis stage and pulmonary function parameters

Pulmonary functional parameters	Stage by CXR (0/I/II/III/IV)		Stage by HRCT (I/II/III/IV)	
	Correlation index	*p* value	Correlation index	*p* value
FVC	−0.073	0.413	−0.125	0.163
FEV1	−0.168	0.06	−0.247	0.006
FEV1/FVC	−0.46	< 0.001	−0.618	< 0.001
DLCO	−0.153	0.139	−0.353	< 0.001
DLCO%	−0.182	0.096	−0.380	< 0.001

Bivariate spearman correlation analysis was performed.

*FVC* forced vital capacity, *FEV1* forced expiratory volume in one second, *DLCO* carbon monoxide diffusing capacity

### Pleural involvement

**Fig. 2:**
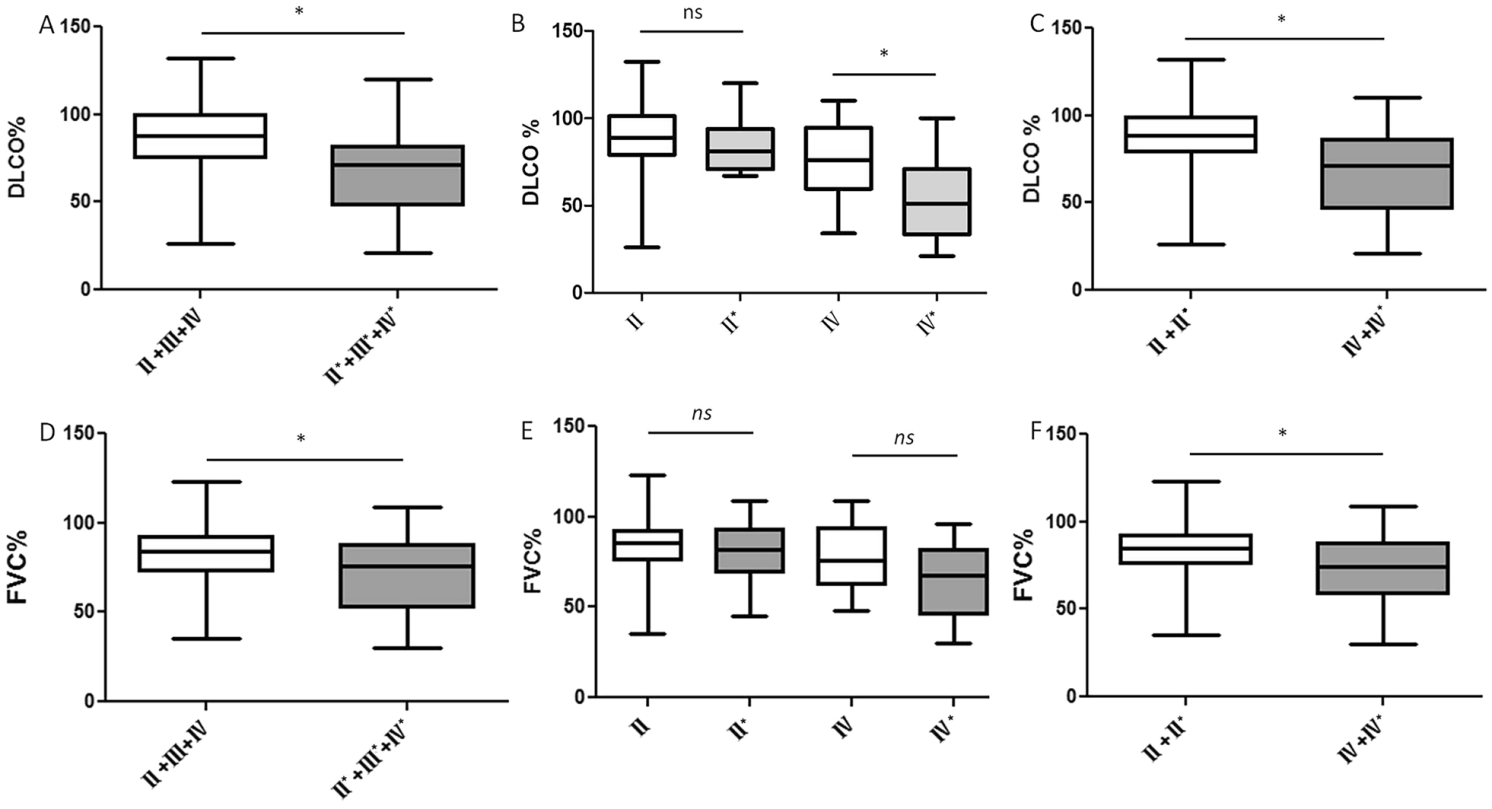
Comparison of DLCO% and FVC% of patients with pleural involvement versus patients without it. **A** Patients with pleural involvement showed significantly poorer DLCO% than patients without it. **B** Patients at stage IV with pleural involvement had significantly poorer DLCO% than patients without it. **C** Patients at stage IV showed significantly poorer DLCO% than patients at stage II. **D** Mean FVC% was significantly poorer in patients with pleural involvement than in patients without it. **E** Although the mean FVC% of patients at stage IV with pleural involvement was less than that of patients at stage IV without pleural involvement, the difference was not statistically significant. **F** Patients at stage IV had significantly poorer FVC% than patients at stage II. ns: non-significant. The difference of two patient groups was analyzed by independent *t*-test. *P* < .05 was considered statistically significant

Of the total 227 patients, HRCT revealed 58 cases (25.6%) of pleural involvement, whereas CXR only found the condition in 17 cases (7.5%). HRCT revealed that 21.4% of the Chinese patients and 34.5% of the American patients had pleural involvement (Additional file 1: Figure S4), including isolated pleural nodules, isolated pleural thickening, and pleural effusion (Additional file 2: Table S2). For patients at stage II, III, or IV, those with pleural involvement had significantly poorer DLCO% (Fig. 2A) and FVC% (Fig. 2D) than the patients without it (All *P* < 0.05). For the patients at stage IV, those with pleural involvement had significantly lower DLCO% than those without it (*P* = 0.0374, Fig. 2B). The patients at stage IV had significantly poorer lung function than the patients at stage II (All *P* < 0.05, Fig. 2C and F). Although the mean FVC% of patients at stage IV with pleural involvement was less than that of patients at stage IV without pleural involvement, the difference was not statistically significant (Fig. 2E).

## Discussion

For more than fifty years, chest x-ray alone has been used to stage the degree of lung involvement [11]. Although the 1999 ATS/ERS/WASOG guidelines do describe that HRCT presentations may be inconsistent with CXR presentations, the staging criteria based on CXR presentations has not been updated because of lacking sufficient clinical evidence. In addition, lung biopsy may identify pulmonary infiltrating lesions in patients with a normal CXR [3, 12]. Moreover, sarcoidosis stage determined from CXR presentations does not correlate with the severity of clinical symptoms and the disease prognosis [7, 8]. Thus, staging sarcoidosis based on CXR presentations may be inadequate in detecting lung involvement and thus may not be as informative as HRCT in directing effective therapies for sarcoidosis.

In most of Chinese ILD centers, HRCT is routinely used for the evaluation of all patients presenting with ILD, including sarcoidosis. HRCT is more sensitive than CXR for the detection of mild adenopathy particularly in subcarinal, anterior, and posterior mediastinal regions. Moreover, CT can reveal lung parenchyma abnormalities not seen on CXR. Use of HRCT may suggest additional diagnostic procedures such as transbronchial lung biopsy and endobronchial ultrasound in patients undergoing evaluation for possible sarcoidosis [13].

Therefore, we believe using HRCT to stage thoracic sarcoidosis may be more accurate and could better support sarcoidosis diagnosis and treatment. In our current study, we reviewed the clinical records of 140 Chinese and 87 American patients with sarcoidosis and staged sarcoidosis based on their chest HRCT and CXR presentations, respectively. Our study found that the rate of consistent stage from CXR and HRCT presentations was only 50.2% (47.1% in Chinese and 55.2% in American patients). The primary reason for the inconsistency was that CXR failed to reveal small lesions because of the limited resolution of CXR. Additionally, CXR could also fail to show hilar/mediastinal lymph node enlargement and lesions involving pleura, which are in the shadow areas of the heart and diaphragm. HRCT can clearly overcome these limitations of CXR. Thus, using HRCT to stage sarcoidosis may more accurately reflect the disease status in the lung, pleura, and intrathoracic lymph nodes than using CXR presentations. However, HRCT can’t totally replace CXR because of issues related to radiation safety and cost.

### Recommendations for an update of pulmonary sarcoidosis staging criteria

The current study suggests that staging sarcoidosis based on CXR presentations may not be accurate. HRCT presentations should be included for staging pulmonary sarcoidosis. Pleural involvement may be associated with sarcoidosis prognosis. Thus, we recommend a routine HRCT and use HRCT presentations for staging thoracic sarcoidosis. Here, we propose thoracic sarcoidosis staging criteria based on HRCT presentations included 5 stages as listed in (Table 4). If pleural involvement occurs, the stage is 0^*^, I^*^, II^*^, III^*^, and IV^*^.

**Table 4:** Sarcoidosis staging criteria based on chest HRCT presentation

Stage	Chest X-ray and HRCT presentations	With pleural involvement
0	No abnormal presentation	0*
I	Bilateral hilar and/or mediastinal lymph node enlargement and without lung infiltration opacities	I*
II	Bilateral hilar and/or mediastinal lymph node enlargement and with reticular, nodular, and patchy opacities	II*
III	Reticular, nodular, and/or patchy opacities and without bilateral hilar and/or mediastinal lymph node enlargement	III*
IV	Pulmonary fibrosis, honeycomb lung, pulmonary bulla, and emphysema	IV*

*stands for with pleural involvement

Compared to the American patients, Chinese patients had substantially higher proportions of stage I and II but lower proportion of stage IV. The Chinese patients presented better pulmonary function at the diagnosis than the American patients, indicating that HRCT may be more widely used in China than in US, and thus more patients with early-stage sarcoidosis could be diagnosed in China.

Zappala and colleagues have suggested that compared with CXR presentations, HRCT presentations appear to be more consistent with pulmonary functional changes [8]. Our current study also showed that sarcoidosis stage determined from HRCT presentations better correlated with pulmonary function parameters than the stage determined from CXR presentations. In particularly, patients with higher disease stage based on HRCT presentations had poorer DLCO and DLCO%, suggesting poorer pulmonary function in these patients. These means the new staging system based on HRCT presentations might recognize severe sarcoidosis patients with poorer prognosis.

Sarcoidosis involving pleura, which usually presents pleural thickening, small pleural nodules, pleural effusion, pneumothorax, chylothorax, and/or hemothorax, has been considered rare for a while [1–3]. In 2005, Szwarcberg and colleagues reported that HRCT identified 25 cases (41%) of pleural involvement in 61 cases of sarcoidosis but CXR only identified 7 cases (11%) [14]. Use of HRCT seems more sensitive than ultrasound in detecting pleural effusion in sarcoidosis [15, 16]. Wang et al. [17] searched the literature databases and found 28 articles which reported 92 cases of sarcoidosis with pleural involvement, including 59 cases of pleural effusion, 29 pleural thickening, 3 pneumothorax, and one pleural nodules. Our current study demonstrated that HRCT identified 58 cases of pleural involvement (58/227, 25.6%) whereas CXR only revealed 17 (17/227, 7.5%). The top three common types of pleural involvement in our study were pleural thickening (26 cases, 11.5%), pleural nodules (18 cases, 7.9%), and pleural effusion (9 cases, 4.0%) that was similar to Huggins’s report (2.8%) [13]. We also found the proportion of pleural involvement in patients with stage IV sarcoidosis increased substantially (23/50, 46%) and patients with pleural involvement (particularly for patients with stage IV) had poorer FVC and DLCO% than patients without it. However, the current sarcoidosis staging criteria does not describe pleural involvement. HRCT can detect pleural involvement more accurately than CXR. Thus, we believe it is necessary to include the presence of pleural involvement as a subtype in thoracic sarcoidosis staging criteria.

## Conclusion

Revised staging criteria based on HRCT presentations included 5 stages with subtypes in the presence of pleural involvement were proposed. Sarcoidosis stage determined based on HRCT presentations appeared to be more accurate than the stage determined from CXR presentations, and can detect different types of pleural involvements. Sarcoidosis with pleural involvement was associated with worse pulmonary diffusion function.

## Supplementary Information

**Additional file 1**. **Figures S1–S4. Figure S1**. Chest X-ray and HRCT images of 3 patients diagnosed as sarcoidosis stage 0 by chest X-ray but stage I, II, and III, respectively, by HRCT presentations. **Figure S2**. Chest X-ray and HRCT images of one patient diagnosed as sarcoidosis stage I by X-ray and stage II by HRCT presentations. **Figure S3**. Chest X-ray and HRCT images of one patient diagnosed as sarcoidosis stage III by chest X-ray and stage II by HRCT presentations. **Figure S4**. HRCT images of one patient showing pleural involvement. A. Pleural nodules. 

**Additional file 2**. **Tables S1 and S2. Table S1**. Reasons for the inconsistent stage determined according to CXR and HRCT presentations. **Table S2**. Pleural involvement in the patients.

## Data Availability

All data generated or analysed during this study are included in this published article.

## Abbreviations

HRCT: High-resolution computed tomography
CXR: Chest X-ray
ILD: Interstitial lung diseases
TB: Tuberculosis
