# Supplementary material for: Chest high-resolution computed tomography can make higher accurate stages for thoracic sarcoidosis than X-ray
Source: BMC Pulm Med. 2022 Apr 16;22:146. doi: 10.1186/s12890-022-01942-y (PMC9013455; doi:10.1186/s12890-022-01942-y)
Supplement: Supplementary file 1 — Additional file 1. Figures S1–S4. Figure S1. Chest X-ray and HRCT images of 3 patients diagnosed as sarcoidosis stage 0 by chest X-ray but stage I, II, and III, respectively, by HRCT presentations. Figure S2. Chest X-ray and HRCT images of one patient diagnosed as sarcoidosis stage I by X-ray and stage II by HRCT presentations. Figure S3. Chest X-ray and HRCT images of one patient diagnosed as sarcoidosis stage III by chest X-ray and stage II by HRCT presentations. Figure S4. HRCT images of one patient showing pleural involvement. A. Pleural nodules. [file 12890_2022_1942_MOESM1_ESM.doc]

**Figures S1-4**

**Figure S1**

**
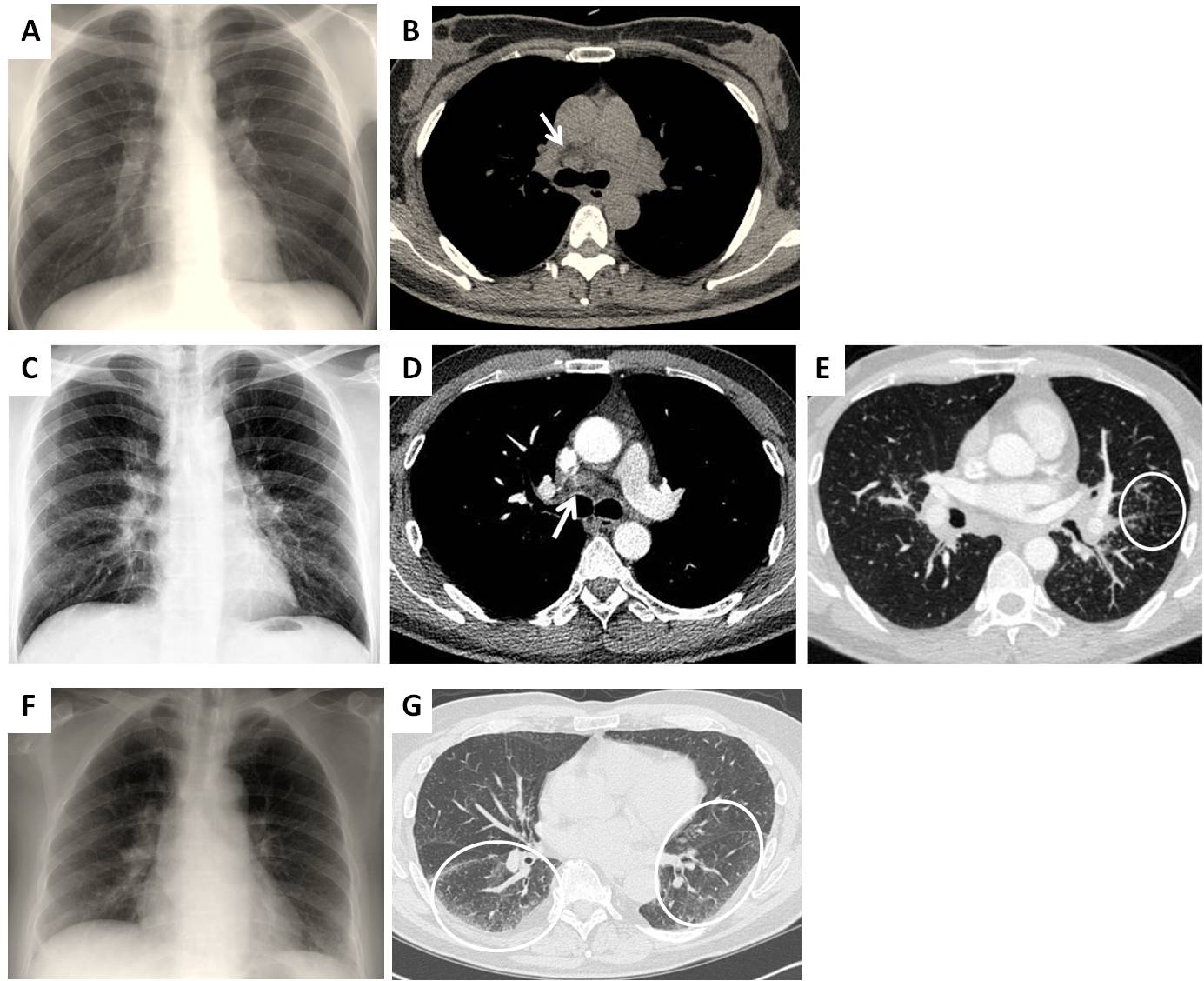
**

**Figure S1.** Chest X-ray and HRCT images of 3 patients diagnosed as sarcoidosis stage 0 by chest X-ray but stage I, II, and III, respectively, by HRCT presentations. A. Case 1 was stage 0 by chest X-ray. B. Case I was Stage I by HRCT. The arrow is pointing to mediastinal lymph node enlargement C. D & E. Case 2 was stage 0 by chest X-ray(C). Stage II by HRCT. The arrow is pointing to mediastinal lymph node enlargement (D). The circle highlights patchy and small nodular opacities (E). F& G. Case 3 was stage 0 by chest X-ray (F). Stage III by HRCT. The circles highlight patchy opacities (G).

**Figure S2**
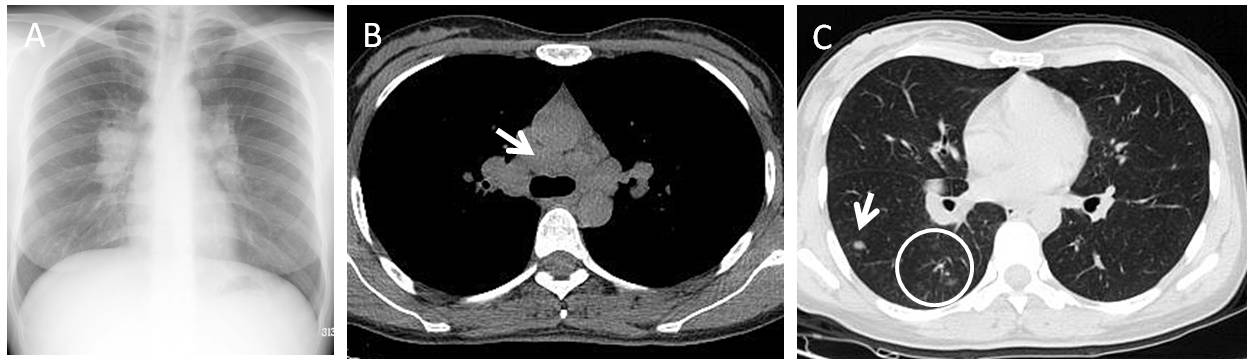


**Figure S2.** Chest X-ray and HRCT images of one patient diagnosed as sarcoidosis stage I by X-ray and stage II by HRCT presentations. A. Stage I by chest X-ray. B. Stage II by HRCT. The arrows are pointing at mediastinal and hilar lymph node enlargement (B) and nodular opacities (C). The circle highlights patchy opacities (C).

**Figure S3
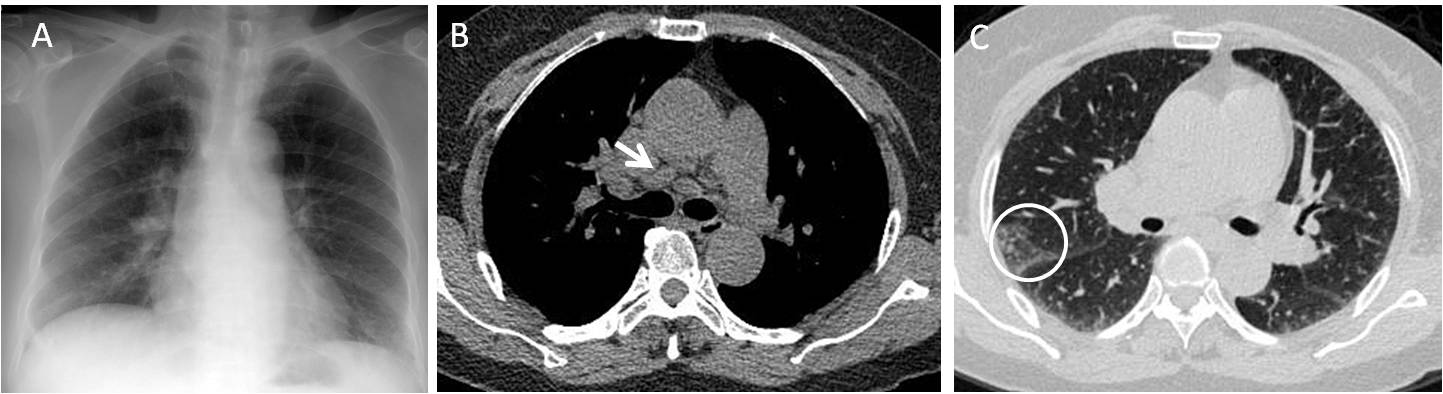
**

**Figure S3.** Chest X-ray and HRCT images of one patient diagnosed as sarcoidosis stage III by chest X-ray and stage II by HRCT presentations. A. Stage III by chest X-ray. B. Stage II by HRCT. The arrow is pointing at mediastinal and hilar lymph node enlargement (B). The circle highlights patchy ground glass opacities underneath the pleura (C).

**Figure S4**


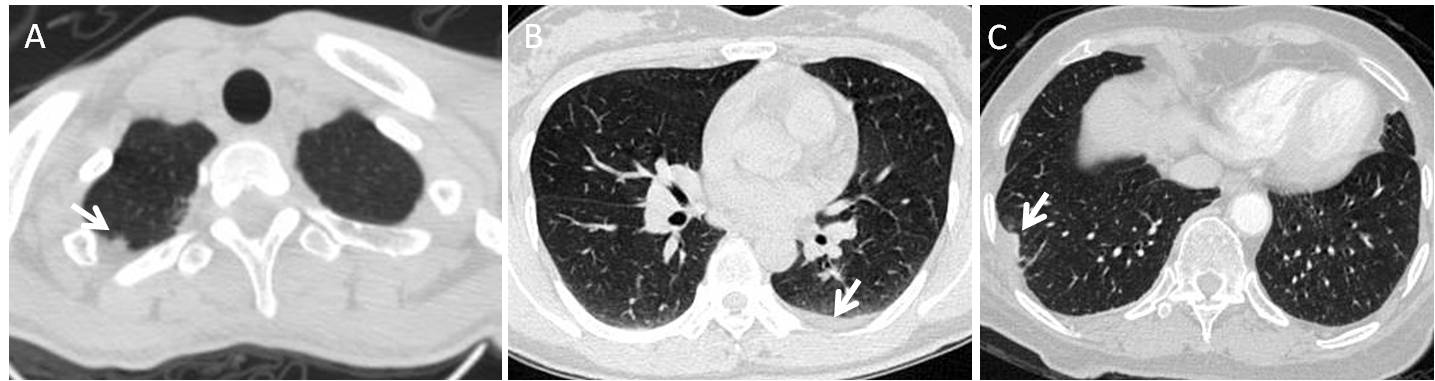
 **Figure S4.** HRCT images of one patient showing pleural involvement. A. Pleural nodules. B. Pleural effusion. C. Pleural thickening. The arrows are pointing at the three types of pleural involvement.
