# Supplementary material for: Chest high-resolution computed tomography can make higher accurate stages for thoracic sarcoidosis than X-ray
Source: BMC Pulm Med. 2022 Apr 16;22:146. doi: 10.1186/s12890-022-01942-y (PMC9013455; doi:10.1186/s12890-022-01942-y)
Supplement: Supplementary file 2 — Additional file 2. Tables S1 and S2. Table S1. Reasons for the inconsistent stage determined according to CXR and HRCT presentations. Table S2. Pleural involvement in the patients. [file 12890_2022_1942_MOESM2_ESM.doc]

**Table S1** Reasons for the inconsistent stage determined according to CXR and HRCT presentations

| **Stage by CXR** | **Number of total cases** | **Number of inconsistent cases by CXR and HRCT** | **Number of inconsistent cases with stage by HRCT** | **Reasons for the inconsistent stage** | | | |
| --- | --- | --- | --- | --- | --- | --- | --- |
|  |  |  |  | **A1** | **A2** | **B1** | **B2** |
| | **Total, n=227** | | | | | | | | | --- | --- | --- | --- | --- | --- | --- | --- | | 0 | 16 | 16 | I, n=2 | 1 | 1 | 0 | 0 | |  |  |  | II, n=11  III, n=3 | 0  0 | 6  0 | 5  3 | 0  0 | | I | 70 | 62 | II, n=62 | 0 | 0 | 50 | 12 | | II | 67 | 5 | IV, n=5 | 0 | 0 | 5 | 0 | | III | 36 | 30 | II, n=23 | 5 | 18 | 0 | 0 | |  |  |  | IV, n=7 | 0 | 0 | 6 | 1 | | IV | 38 | 0 | NA | NA | NA | NA | NA | | Total | 227 | 113 |  | 6 | 25 | 69 | 13 | | | | | | | | |
| Chinese patients, n = 140 | | | | | | | |
| 0 | 9 | 9 | I, n=2 | 1 | 1 | 0 | 0 |
|  |  |  | II, n=7 | 0 | 5 | 2 | 0 |
| I | 54 | 48 | II, n=48 | 0 | 0 | 38 | 10 |
| II | 56 | 0 | NA | NA | NA | NA | NA |
| III | 19 | 17 | II, n=15 | 3 | 12 | 0 | 0 |
|  |  |  | IV, n=2 | 0 | 0 | 2 | 0 |
| IV | 2 | 0 | NA | NA | NA | NA | NA |
| Total | 140 | 74 |  | 4 | 18 | 42 | 10 |
| American patients, n = 87 | | | | | | | |
| 0 | 7 | 7 | II, n=4 | 0 | 1 | 3 | 0 |
|  |  |  | III, n=3 | 0 | 0 | 3 | 0 |
| I | 16 | 14 | II, n=14 | 0 | 0 | 12 | 2 |
| II | 11 | 5 | IV, n=5 | 0 | 0 | 5 | 0 |
| III | 17 | 13 | II, n=8 | 2 | 6 | 0 | 0 |
|  |  |  | IV, n=5 | 0 | 0 | 4 | 1 |
| IV | 36 | 0 | NA | NA | NA | NA | NA |
| Total | 87 | 39 |  | 2 | 7 | 27 | 3 |

NA: not applicable.

A1: hilar lymph node enlargement was missed from anteroposterior CXR; A2: mediastinal lymph node enlargement in the shadow areas of the heart was missed from anteroposterior CXR; B1: small pulmonary nodules, mild infiltrating, thin patchy, and/or linear opacities were missed from CXR because the low resolution of CXR; B2: pulmonary nodules, infiltrating, patchy, and/or linear opacities were missed from CXR because the lesions were posterior to the heart, at the lung apexes, at the lung bases, or near the diaphragm.

**Table S2 Pleural involvement in the patients**

| **Stage by HRCT** | | **Number of cases** | **Pleural involvement** | | | | | | | |
| --- | --- | --- | --- | --- | --- | --- | --- | --- | --- | --- |
|  |  |  | Pleural nodules | | Pleural thickening | | | Pleural effusion | | Total (n) |
| **Total, n = 227** | | | | | | | | | | |
| 0 | 0 | | | 0 | | 0 | 0 | | 0 | |
| I | 10 | | | 0 | | 0 | 0 | | 0 | |
| II | 158 | | | 13 | | 15 | 6 | | 34 | |
| III | 9 | | | 0 | | 2 | 1 | | 3 | |
| IV | 50 | | | 3 | | 18 | 2 | | 23 | |
| Total | 227 | | | 16 | | 35 | 9 | | 60(26.4%) | |
| Chinese patients, n = 140 | | | | | | | | | | |
| 0 | | 0 | 0 | | 0 | | | 0 | | 0 |
| I | | 8 | 0 | | 0 | | | 0 | | 0 |
| II | | 126 | 8 | | 12 | | | 6 | | 26 |
| III | | 2 | 0 | | 2 | | | 0 | | 2 |
| IV | | 4 | 1 | | 1 | | | 0 | | 2 |
| Total | | 140 | 9 | | 15 | | | 6 | | 30 (21.4%) |
| American patients, n = 87 | | | | | | | | | | |
| 0 | | 0 | 0 | | 0 | | | 0 | | 0 |
| I | | 2 | 0 | | 0 | | | 0 | | 0 |
| II | | 32 | 5 | | 3 | | | 0 | | 8 |
| III | | 7 | 0 | | 0 | | | 1 | | 1 |
| IV | | 46 | 2 | | 17 | | | 2 | | 21 |
| Total | | 87 | 7 | | 20 | | | 3 | | 30 (34.5%) |

**Figures S1-4**

**Figure S1**

**
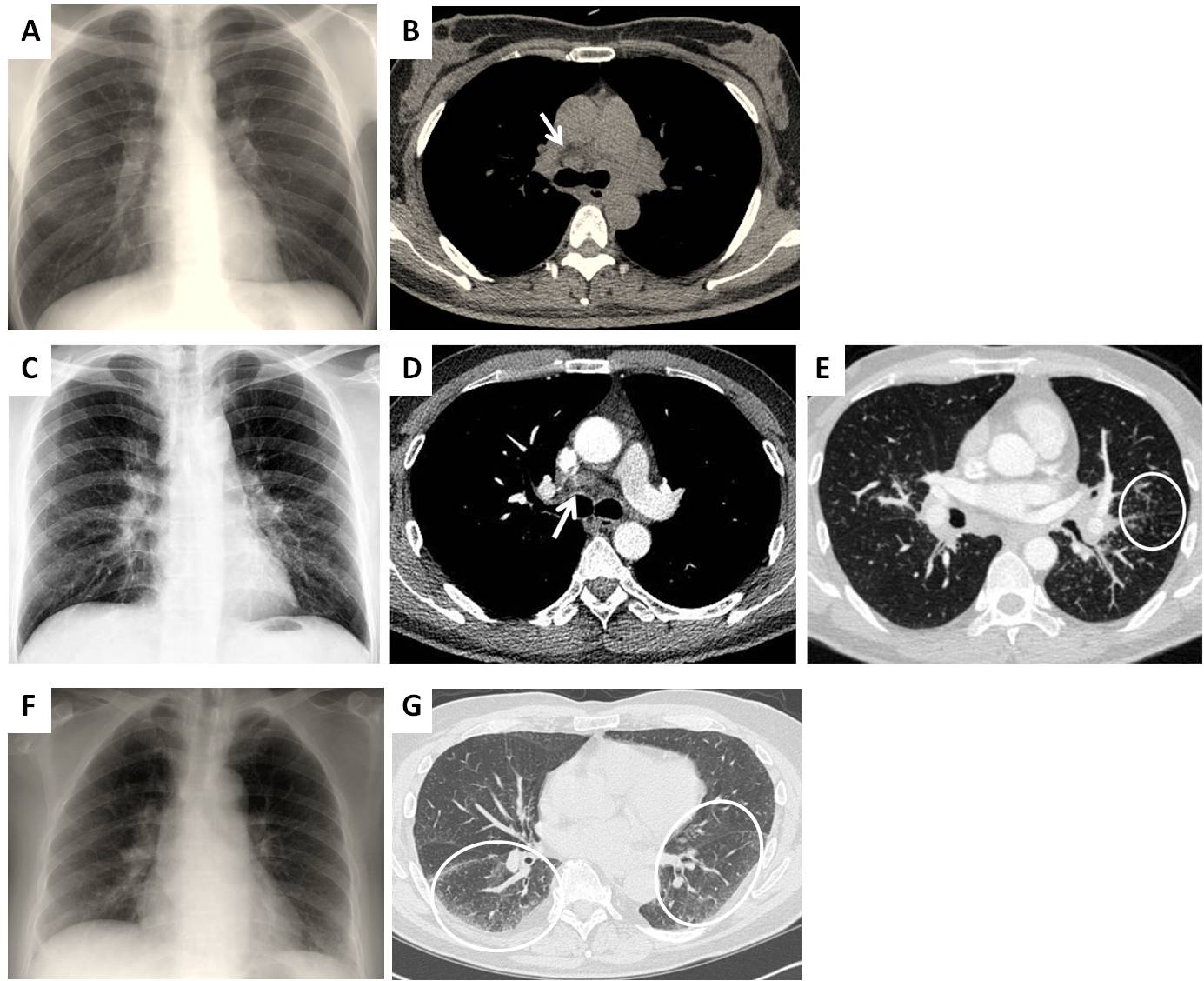
**

**Figure S1.** Chest X-ray and HRCT images of 3 patients diagnosed as sarcoidosis stage 0 by chest X-ray but stage I, II, and III, respectively, by HRCT presentations. A. Case 1 was stage 0 by chest X-ray. B. Case I was Stage I by HRCT. The arrow is pointing to mediastinal lymph node enlargement C. D & E. Case 2 was stage 0 by chest X-ray(C). Stage II by HRCT. The arrow is pointing to mediastinal lymph node enlargement (D). The circle highlights patchy and small nodular opacities (E). F& G. Case 3 was stage 0 by chest X-ray (F). Stage III by HRCT. The circles highlight patchy opacities (G).

**Figure S2**
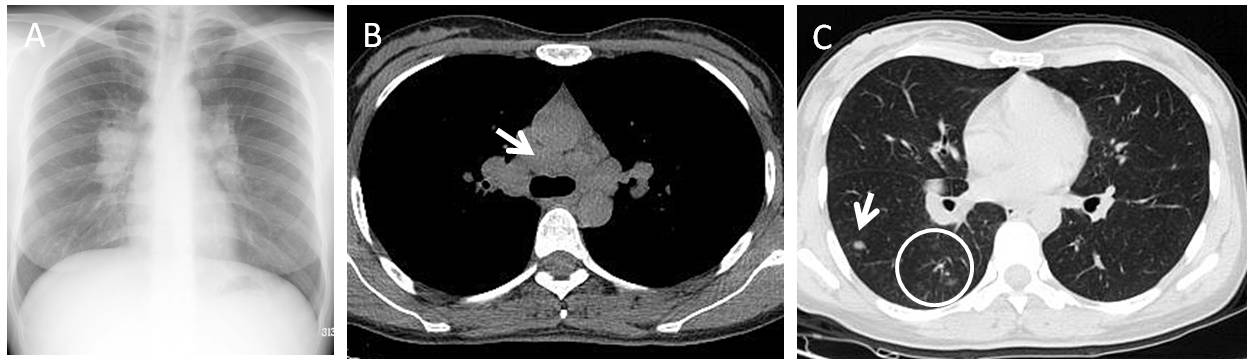


Chest X-ray and HRCT images of one patient diagnosed as sarcoidosis stage I by che **Figure S2.** st X-ray and stage II by HRCT presentations. A. Stage I by chest X-ray. B. Stage II by HRCT. The arrows are pointing at mediastinal and hilar lymph node enlargement (B) and nodular opacities (C). The circle highlights patchy opacities (C).

**Figure S3
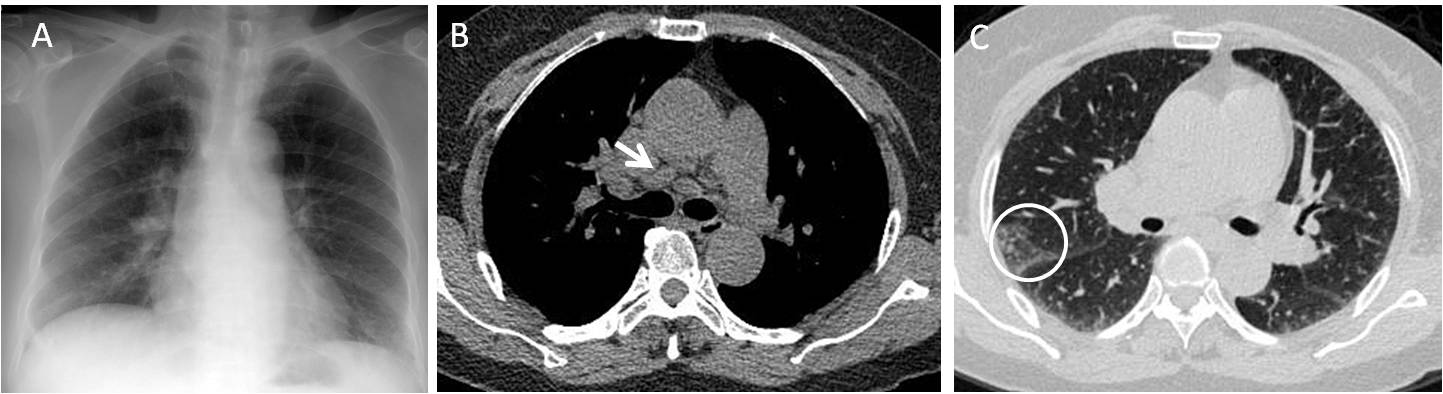
**

**Figure S3.** Chest X-ray and HRCT images of one patient diagnosed as sarcoidosis stage III by chest X-ray and stage II by HRCT presentations. A. Stage III by chest X-ray. B. Stage II by HRCT. The arrow is pointing at mediastinal and hilar lymph node enlargement (B). The circle highlights patchy ground glass opacities underneath the pleura (C).

**Figure S4**


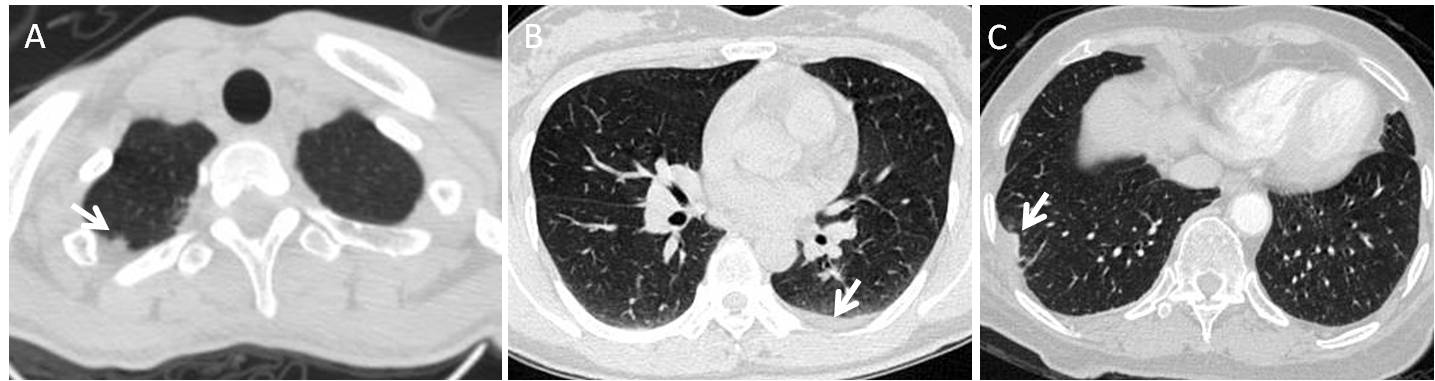
 **Figure S4.** HRCT images of one patient showing pleural involvement. A. Pleural nodules. B. Pleural effusion. C. Pleural thickening. The arrows are pointing at the three types of pleural involvement.
